# Analysis of pharmacotherapeutic approaches for multiple myeloma and correlated renal and pulmonary impairments: a retrospective real-world registry study in the Greater Gulf Region (REPAIR Study)

**DOI:** 10.3389/fonc.2025.1547138

**Published:** 2025-05-09

**Authors:** Ayman Alhejazi, Ahmad Alhuraiji, Abdulnaser Nourallah, Abdulrahman Alshehri, Binyam Usman, Ghada ElGohary, Hafiz Malhan, Ibraheem Motabi, Khalil Al Farsi, Mohammed Alshuaibi, Mohanad Diab, Mustaqeem Siddiqui, Ruba Yasin Taha, Tarek Abouzeid, Wesam Ahmed, Ahmed Ramadan Ali, Rasha Ghonema, Sana Faysal Elkhazin, Yousra Moussa, Abdullah M. Alrajhi, Magdy Rabea, Yahia Aktham, Nesreen Bawazeer, Ali Ahmed Ali, Mohamed Zahir Chouikrat

**Affiliations:** ^1^ Division of Adult Hematology, Department of Oncology, King Abdulaziz Medical City, Riyadh, Saudi Arabia; ^2^ Department of Hematology, Kuwait Cancer Control Center, Kuwait, Kuwait; ^3^ Hematology/Medical Oncology Department, Almana General Hospital, Alkhobar, Saudi Arabia; ^4^ Hematology/Oncology Department, Aseer Central Hospital, Abha, Saudi Arabia; ^5^ Department of Oncology, King Faisal Specialist Hospital and Research Center, Jeddah, Saudi Arabia; ^6^ Department of Medicine, Division of Oncology/Hematology, College of Medicine, King Saud University Medical City, Riyadh, Saudi Arabia; ^7^ Department of Adult Hematology, Prince Mohammed bin Nasser Hospital, Jazan, Saudi Arabia; ^8^ Adult Hematology and Bone Marrow Transplant Department, Comprehensive Cancer Center at King Fahad Medical City, Riyadh, Saudi Arabia; ^9^ Adult Hematology Department, Alfaisal University, Riyadh, Saudi Arabia; ^10^ Department of Hematology, Sultan Qaboos University Hospital, Muscat, Oman; ^11^ Adult Hematology and Oncology Divisions, Department of Medicine, King Abdul-Aziz Hospital, Alahsa, Saudi Arabia; ^12^ Hemato-oncology Department, Burjeel Hospital, Abu Dhabi, United Arab Emirates; ^13^ Hematology and Oncology Division at Sheikh Shakhbout Medical City (SSMC), Abu Dhabi, United Arab Emirates; ^14^ Department of Hematology-Bone Marrow Transplantation, National Centre for Cancer Care and Research (NCCCR), Hamad Medical Corporation (HMC), Doha, Qatar; ^15^ Internal Medicine Department, Almouwasat Hospital, Dammam, Saudi Arabia; ^16^ Oncology Institute, Cleveland Clinic Abu Dhabi, Abu Dhabi, United Arab Emirates; ^17^ Oncology Institute, Cleveland Clinic Florida, Florida, FL, United States; ^18^ Clinical Pharmacy Department, King Fahad Medical City, Riyadh, Saudi Arabia; ^19^ Department of Pharmacy Practice, College of Pharmacy, AlFaisal University, Riyadh, Saudi Arabia; ^20^ Medical Affairs Department, Sanofi, Jeddah, Saudi Arabia; ^21^ Medical Affairs Department, Sanofi, Dubai, United Arab Emirates

**Keywords:** multiple myeloma, relapsed/refractory, Gulf Region, real-world, isatuximab

## Abstract

**Background:**

Multiple myeloma (MM) is a plasma cell malignancy with significant unmet medical needs, particularly in the treatment of relapsed and refractory disease. This study aims to describe the disease characteristics, various treatment regimens, and outcomes among patients with Relapsed/Refractory Multiple Myeloma (RRMM) in the Greater Gulf region.

**Methods:**

A regional, retrospective study was conducted in Gulf countries to collect real-world data from the medical records of 148 patients with RRMM who relapsed 1–3 times in the past two years before the data collection period (July 2022 and February 2023).

**Results:**

The mean age of the study population was 59.4 years, and 64.2% of the participants were male. The VRd regimen (Bortezomib, Lenalidomide, and Dexamethasone) was the most frequent first-line therapy among transplant-ineligible patients (40.2%) and the most common induction and consolidation regimen (43.9% and 66.7%, respectively) in transplant-eligible patients. Meanwhile, Rd (Lenalidomide and Dexamethasone) was the most common maintenance regimen (75%). DKd (Daratumumab, Carfilzomib, and Dexamethasone), KPd (Carfilzomib, Pomalidomide, and Dexamethasone), and PVd (Pomalidomide, Bortezomib, and Dexamethasone) were the most widely used second, third, and fourth treatment lines, respectively (16.6%, 9.2%, and 12.5%). About 52.7% of patients were eligible for stem cell transplantation (SCT), and among them, a complete response (CR) was achieved in 47.7%. Furthermore, CR and very good partial remission rates decreased across all treatment lines. Renal impairment decreased across different treatment lines, from 23.6% in the first line to 6.3% in the fourth line. In contrast, respiratory complications demonstrated the highest incidence (>18%) in the 3rd and 4th treatment lines. Moreover, refractoriness to treatment increased from 1.3% in the first line to 34.6% in the fourth treatment line. Additionally, isatuximab was incorporated into 80%, 15%, and 5% of the regimens administered as second-, third-, and fourth-line treatments, respectively.

**Conclusion:**

This study provides valuable insights into the real-world management and treatment choices for RRMM, including the utilization of SCT and novel therapies such as isatuximab.

## Introduction

Multiple myeloma (MM) is a rare plasma cell malignancy arising from the bone marrow (BM) (1). These plasma cells produce abnormal monoclonal immunoglobulins that target various organs, resulting in multisystem complications including bone lesions, renal impairment, anemia, and other associated morbidities (2–5).

MM ranks as the second most common hematologic malignancy, following lymphoma, accounting for approximately 1% of all cancers and around 10% of hematologic malignancies (6). Despite the availability of various treatment options for MM, such as targeted therapies or chemotherapy, patients frequently experience multiple relapses or develop resistance to medical interventions (7). Currently, four drug classes are employed for MM treatment including proteasome inhibitors (PI: bortezomib, carfilzomib, and ixazomib), immunomodulatory drugs (IMiDs: thalidomide, lenalidomide, and pomalidomide), monoclonal antibodies (mAbs), including CD38-targeted mAbs (daratumumab and isatuximab) and SLAMF7-directed immunostimulatory antibody (elotuzumab), and the selective inhibitor of nuclear export (Selinexor) (8, 9). Isatuximab is a humanized IgG1 monoclonal antibody that targets CD38, a cell surface glycoprotein expressed on the surface of MM cells (10). It is approved for the treatment of relapsed or refractory multiple myeloma (RRMM) in combination with either pomalidomide and dexamethasone (11) or carfilzomib and dexamethasone (12). Additionally, stem cell transplantation (SCT) is considered a valuable treatment option for MM, primarily due to its ability to prolong progression-free survival (PFS) and overall survival (OS). offering a good chance for a long-lasting response (8, 13, 14). The selection of first-line therapeutic options involves various combination patterns determined by factors such as the patient’s disease status, transplant eligibility, associated comorbidities, and the functionality of the renal, hepatic, and pulmonary systems. They can also be used in the treatment of RRMM. Specifically, the addition of anti-CD-38 monoclonal antibodies to subsequent treatment regimens of IMiDs (or PIs) and dexamethasone could offer a promising treatment option for patients with RRMM (15).

Notably, lung and renal complications, among others, significantly impact patients’ quality of life and disease prognosis. A substantial proportion of MM patients exhibit chronic obstructive pulmonary disease (COPD), bronchial asthma (BA), or renal impairment at the time of diagnosis or during the disease course (3, 4).

Renal impairment is highly prevalent among MM patients, ranging from 25% to 60% (2). Its complexity lies in the heterogeneous nature of lesions, which are dependent on specific renal sites (5, 16). Nephrotoxic processes, including dehydration, hypercalcemia, immunoglobulin deposition, and infections, can exacerbate renal damage (17). Also, real-world data by Rice et al. revealed that a significant proportion of MM patients (up to 15%) suffer from lung diseases, including COPD or BA (4). Additionally, exposure to toxic medications contributes to the deterioration of both renal and pulmonary functions in MM patients (18).

As defined by the International Myeloma Working Group (IMWG), RRMM is characterized by non-responsiveness or progression within 60 days of the last treatment in patients who previously achieved a minimal response or higher on prior therapy (19). Despite the potential for long-term disease remission, the natural course of MM often leads to relapse after initial treatments (20, 21).

In the Greater Gulf region, there is a lack of real-world data on MM-associated morbidities, treatment patterns (including various treatment lines and combination regimens) in RRMM, and treatment outcomes. Therefore, we aim to fill this void by providing insights into the current treatment landscape for MM, the status of renal and pulmonary impairment among RRMM patients in the region, and the prevalence of lenalidomide-refractory MM patients to enhance understanding and management strategies for MM in this region.

## Methods

### Study design and setting

This study was conducted in tertiary-level care centers in Saudi Arabia, the United Arab Emirates (UAE), Kuwait, and Qatar. The study was a regional retrospective study that collected real-world and epidemiological data from MM patient records, including electronic, paper charts, or any other documentation, in the included countries. The diagnosis of patients with MM was established following the International Myeloma Working Group (IMWG) criteria for the diagnosis of MM (22). The study was performed following the principles of the Declaration of Helsinki. At each center, the protocol was approved by the institutional review board (IRB) or ethics committee. Also, the study protocol was registered and published (International Registered Report: DERR1-10.2196/49861) (23). Due to the retrospective nature of the study, informed consent from patients was not required.

### Eligibility criteria

Eligible patients were male or female adults (≥18 years old) diagnosed with RRMM who had experienced a relapse at least once and up to three times within the last two years preceding the data collection date. Patients should have received 1–3 prior lines of treatment within the 2 years preceding data collection. Complete patient medical records from the initial MM diagnosis to the date of death or medical abstraction were also required.

Conversely, exclusion criteria involve patients who were not receiving any treatment for MM or those newly diagnosed. Patients with a history of other malignancies or current pregnancy were also excluded. Additionally, patients with end-stage renal disease (ESRD) were excluded from participation in the study.

### Study objectives

The primary objective of this study was to provide a comprehensive description of the characteristics and treatment landscape of patients with RRMM in the Greater Gulf region. The key secondary objectives included MM disease history; the percentage of SCT-eligible patients; time to progression (TTP); duration of response (DoR); response to various lines of treatment; the frequency of relapse and refractoriness, including lenalidomide-refractory patients; the prevalence of renal impairment and respiratory complications, the rate of improvement across all the treatment lines; and the minimal residual disease (MRD) status.

Renal impairment in this study was defined based on clinical documentation at diagnosis or during treatment, including elevated serum creatinine levels, acute kidney injury (AKI), chronic kidney disease (CKD), or acute on top of CKD. It also included myeloma cast nephropathy and progressive deterioration of renal function.

Respiratory complications were defined as the proportion of MM patients with asthma and/or COPD.

### Data sources, collection, and monitoring at site level

The data sources for this study included original or certified copies of various medical records related to MM patients, such as hospital records, office charts, evaluation checklists, laboratory reports, and radiology reports. Existing medical records at each site served as the primary source of data for extracting the required information for eligible patients.

Data collection, validation, and quality control involved computerized handling with pre-programmed validation rules outlined in the Data Validation Plan (DVP). The system automatically generated queries based on these rules, and additional queries were raised through manual or medical reviews. Site staff were responsible for resolving these queries through the Electronic Data Capture (EDC) system.

Monitoring and data quality control were conducted at the site level for 50% of randomly chosen active sites across the country. Qualified designated personnel in each country performed data quality control through site monitoring and/or phone quality control, following the detailed methodologies outlined in the study manual.

Data collection, monitoring, and quality control were performed by personnel from the RAY-contract research organization, an independent third-party entity.

### Statistical analysis and sample size calculation

Quantitative variables were reported using mean, median, and standard deviation (SD), while qualitative variables were presented as counts (n) and absolute percentages (%) for each study variable. Pearson’s Chi-square test was used to investigate the association between asthma, COPD, or renal impairment across various treatment lines.

Given the primarily descriptive nature of the analyses, the sample size was adjusted to estimate percentages with acceptable precision, considering the challenges posed by data scarcity in the Greater Gulf Region. The maximum variance occurs at 50%, leading to the estimation of the sample size based on this worst-case scenario. A minimum of 150 patients were required to achieve an observed percentage of 50% with an absolute precision of 8% and a 95% confidence interval (CI). Considering potential exclusions of 10% due to missing values or unmet inclusion/exclusion criteria, approximately 170 patients were needed for enrollment in the study.

The absence of a literature review within the Greater Gulf Region left no established benchmarks for sample size calculation in this context. While adhering to the standard proportion formula, the authors acknowledged the significance of statistical power. However, given the exploratory nature of this ‘pilot’ study, initial expectations were adjusted as the focus was on paving the road for future studies. The overarching goal was to explore current management practices and address the existing gap in this region. Should circumstances require, the study would continue with the established formula, maintaining the worst-case analysis with an estimated proportion of 50% for any qualitative variable ( $n=\frac{z^{2}\;\times \;p\left(1-p\right)}{d^{2}}$ ).

## Results

### Baseline characteristics

Between July 2022 and February 2023, 148 MM patients of 153 assessed for eligibility were enrolled and included in the study analysis. All included patients (n = 148) had received at least one line of therapy; 139, 54, and 16 patients had received two, three, and four lines of therapy, respectively (**Supplementary Figure S1**).

The patients’ baseline characteristics and demographics are presented in **Table 1**. The mean age (SD) at baseline was 59.5 (12) years, 64.2% of the patients were males, and 45.3% were Saudi. Additionally, the majority of the population (n = 88) were identified as Arabs.

**Table 1:** Patients’ baseline characteristics.

Patients’ Characteristics	Values
Nationality, N (%)	
KSA	67 (45.3%)
UAE	6 (4.1%)
Kuwaiti	13 (8.8%)
Not available	2 (1.4%)
Others	60 (40.5%)
Gender, N (%)	
Male	95 (64.2%)
Female	53 (35.8%)
**Age, mean (SD)**	59.5 (12)
Race, N (%)	
White	2 (1.3%)
Asian	25 (16.8%)
Caucasian	20 (13.5%)
Black or African American	6 (4%)
NA	7 (4.7%)
Others:	88 (59.4%)
*Arab*	*85 (96.5%)*
*Persian*	*1 (1.1%)*
*Punjabi*	*2 (2.2%)*

*KSA: Kingdom of Saudi Arabia, UAE: United Arab Emirates, SCT: Stem cell transplantation, NA: Not available.*

The median age at diagnosis was 56 years (range: 18-84), and the median duration of the disease at the time of data collection was 35 months (range: 2-246). Moreover, 39.9% and 26.4% of the patients had stage III and II disease, respectively, according to the International Staging System (ISS) (**Supplementary Table S1**).

### Treatment regimens

The VRd (Bortezomib, Lenalidomide, and Dexamethasone) regimen was the most common first-line therapy among MM patients not receiving SCT (40.2%) and the most frequently administered induction and consolidation regimen among patients undergoing SCT (43.9% and 66.7%, respectively). Meanwhile, Rd (Lenalidomide and Dexamethasone) was the most common maintenance regimen (75%). The most common 2^nd^ line treatment combinations were DKd (Daratumumab, Carfilzomib, and Dexamethasone) (16.6%), DVd (Bortezomib, Daratumumab, and Dexamethasone) (12.2%), followed by DRd (Daratumumab, Lenalidomide, Dexamethasone) or Isa-Pd (Isatuximab, Pomalidomide, and Dexamethasone) regimens (10.8% for each). Different triple regimens were used as third- and fourth-line therapy; the most frequently administered third-line regimen was KPd (Carfilzomib, Pomalidomide, and Dexamethasone) (9.2%). PVd (Pomalidomide, Bortezomib, and dexamethasone) was the most widely used 4^th^ line therapy (12.4%). More data on treatment regimens are available in **Table 2**.

**Table 2:** Treatment regimens used among the study population.

Treatment regimens	N (%)
1st line regimens (No SCT)*	
Bortezomib, Lenalidomide, Dexamethasone + (Denosumab, n=2)	33 (40.2%)
Bortezomib, Lenalidomide, Dexamethasone, CYC + (Radiation, and Zoledronic acid, n=2)	6 (7.3)
Bortezomib, Thalidomide, Dexamethasone	2 (2.4%)
CYC, Bortezomib, Dexamethasone + (Thalidomide, Zoledronic acid, ixazomib, Radiation, n=4)	17 (20.7%)
Daratumumab, Lenalidomide, Dexamethasone + (Radiation, Denosumab, n=2)	10 (12.2%)
Other Regimens	14 (17.1 %)
1st line treatment induction regimens for SCT patients	
Bortezomib, CYC, Dexamethasone, Lenalidomide + (Thalidomide, n=1)	6 (9.1%)
Bortezomib, CYC, Dexamethasone	16 (24.2%)
Daratumumab, Bortezomib, Dexamethasone, Lenalidomide	3 (4.5%)
Dexamethasone, Bortezomib, Lenalidomide + (Radiation, n=3)	29 (43.9%)
Other regimens	12 (18.2%)
1st line treatment consolidation regimens for SCT patients	
Bortezomib, CYC, Dexamethasone	1 (11.1%)
Bortezomib, Lenalidomide, Dexamethasone + (Melphalan, n=1)	6 (66.7%
Dexamethasone, Daratumumab, Lenalidomide	1 (11.1%)
Melphalan, Lenalidomide, Dexamethasone	1 (11.1%)
1st line treatment maintenance regimens for SCT patients	
Bortezomib, Dexamethasone	4 (14.3%)
Lenalidomide, Dexamethasone	21 (75%)
Other regimens	3 (10.7%)
2nd line treatment regimens	
Bortezomib, CYC, Dexamethasone	5 (3.6%)
Bortezomib, Daratumumab, Dexamethasone	17 (12.2%)
Bortezomib, Lenalidomide, Dexamethasone + (Denosumab. n=1)	8 (5.8%)
Carfilzomib, Lenalidomide, Dexamethasone + (Radiation, n=1)	9 (6.5%)
Carfilzomib, Daratumumab, Dexamethasone + (Melphalan, and/or Radiation=3, Venetoclax= 5)	23 (16.6%)
Carfilzomib, Dexamethasone, Pomalidomide	3 (2.2%)
Daratumomab, Pomalidomide, Dexamethasone	9 (6.5%)
Daratumumab, Dexamethasone	3 (2.2%)
Daratumumab, Lenalidomide, Dexamethasone	15 (10.8%)
Isatuximab, Pomalidomide, Dexamethasone	16 (11.5%)
Lenalidomide, Dexamethasone + (Melphalan, n=1)	5 (3.6%)
Other regimens	26 (18.7%)
3rd line treatment regimens	
Bortezomib + (Dexamethasone, n=1)	3 (5.5%)
Carfilzomib, Daratumumab, Dexamethasone	4 (7.4%)
Carfilzomib, Pomalidomide, Dexamethasone	5 (9.2%)
Daratumumab, Pomalidomide, Dexamethasone,	4 (7.4%)
Dexamethasone, CYC, Pomalidomide + (Bortezomib, n=1)	3 (5.5%)
Dexamethasone, Lenalidomide, Daratumumab	4 (7.4%)
Dexamethasone, Pomalidomide, Isatuximab	3 (5.5%)
Melphalan + (Prednisolone, Morphine, Dexamethasone, ASCT, n=4)	3 (5.5%)
Dexamethasone, Cyclophosphamide, Carfilzomib	4 (7.4%)
NA	1 (1.8%)
Other regimens	20 (37%)
4th line treatment regimens	
Atoplozoid, Cisplatin, CYC, Dexamethasone	1 (6.2%)
Dexamethasone, Bortezomib	1 (6.2%)
Dexamethasone, Bortezomib, Bendamustine	1 (6.2%)
Dexamethasone, Bortezomib, Pomalidomide	2 (12.4%)
Dexamethasone, Carfilzomib, Lenalidomide	1 (6.2%)
Dexamethasone, CYC, Carfilzomib	1 (6.2%)
Dexamethasone, CYC, Pomalidomide	1 (6.2%)
Dexamethasone, Pomalidomide, Carfilzomib	1 (6.2%)
Dexamethasone, Pomalidomide, Isatuximab	1 (6.2%)
Etoposide, CYC, Doxorubicin /Cisplatin, Dexamethasone, Bortezomib, Radiotherapy, Pomalidomide	1 (6.2%)
Prednisone, Melphalan, Bortezomib	1 (6.2%)
NA	4 (25%)

*N= 82

*CYC, cyclophosphamide; SCT, stem cell transplantation; NA, not available; ASCT, autologous stem cell transplant.*

### Stem cell transplantation among the study participants

Among the study population, 78 patients (52.7%) were considered eligible for SCT. The majority of the patients who were not eligible for SCT were either elderly (43.3%) or refused the procedure (27.5%). SCT was performed in 85.9% of eligible patients, with autologous SCT being the most common type (84.6%). The stem cells were sourced from either peripheral blood (61.5%), bone marrow (19.2%), or cord blood (1.3%). Following the transplant, a complete response (CR) was achieved in 47.76% of patients, and very good partial response (VGPR) and partial response (PR) were observed in 14.93% and 11.94% of patients, respectively. Additionally, 70.6% of patients who underwent MRD testing post-SCT showed MRD negativity (**Supplementary Table S2**).

### The proportion of relapses and refractoriness

After the 1^st^, 2^nd^, and 3^rd^ lines of treatment, 98.6%, 83.8%, and 65.3% experienced relapse, and 1.3%, 16.1%, and 34.6% experienced refractoriness to the treatment, respectively (**Supplementary Table S3**).

Among patients receiving their first, second, third, and fourth lines of treatment, 28.4%, 25.9%, 24.1%, and 18.8% were lenalidomide-refractory, respectively (**Table 3**).

**Table 3:** Number of lenalidomide-refractory patients in each treatment line.

Lines of treatment	N (%)
1^st^ line (post 1^st^ relapse), N=148	42 (28.4)
2^nd^ line, N=139	36 (25.9)
3^rd^ line, N=54	13 (24.1)
4^th^ line, N=16	3 (18.8)

### Time to disease progression among the study participants

The overall median TTP was 36.9 months (range: 20.4-244.8). Besides, the median TTP was 23.6 (range: 1.5- 175.6), 11.3 (range: 4- 38.8), 6.9 (range: 3.4- 50), and 2.5 (range: 0.2- 12.8) months from the initiation of the 1st, 2nd, 3rd, and 4th lines to the date of progression, respectively. Furthermore, the overall median DoR among the participants was 23.8 months (range: 20.3-238.7). The median DoR was 17.2 (range: 0.03- 171.8), 7.45 (range: 0.1-65.1), and 4.4 (range: 0.5-44.7) months for patients receiving the 1st, 2nd, and 3rd lines of treatment, respectively (**Supplementary Table S4**).

**Figure 1:**
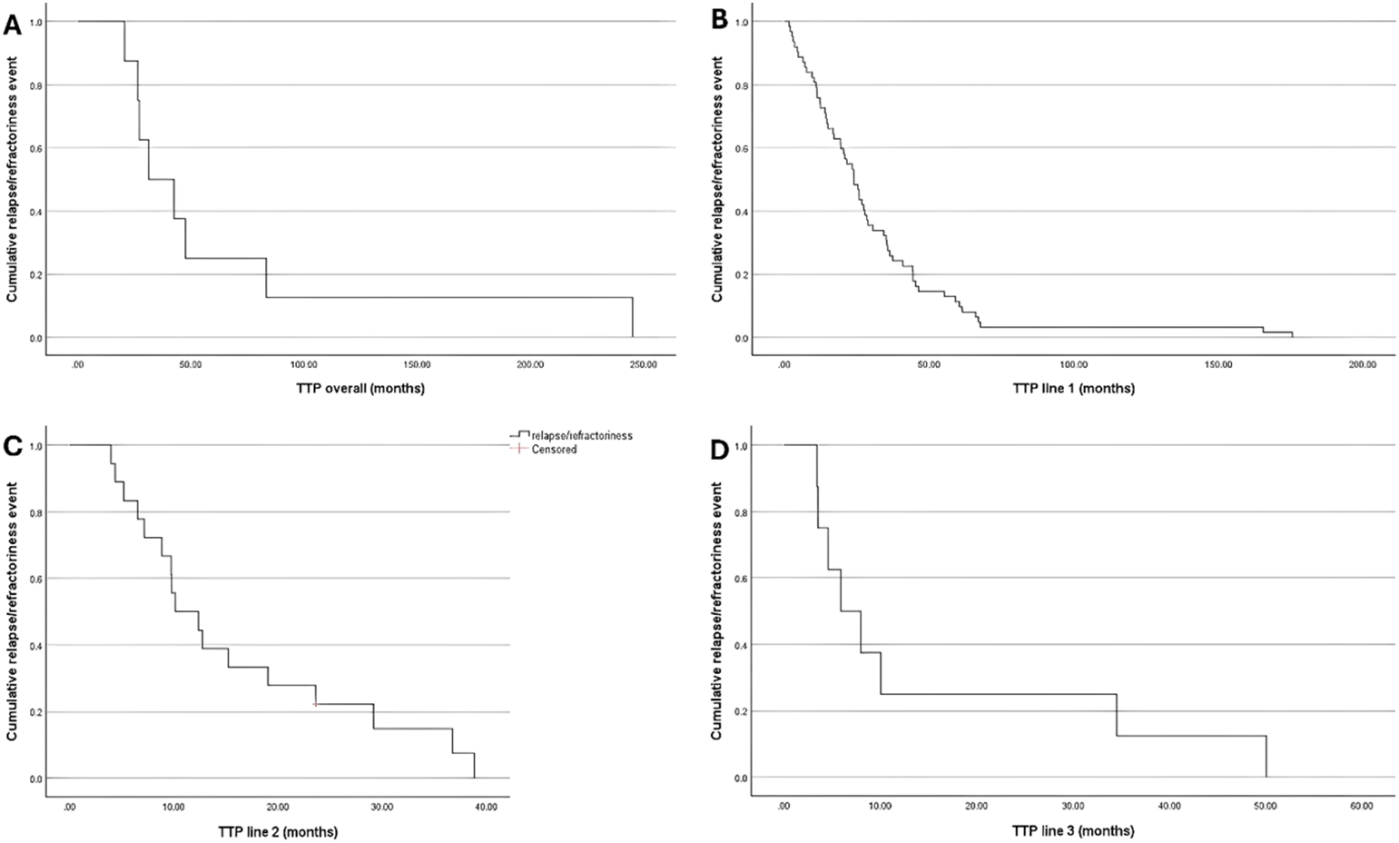
Kaplan-Meir Curve for TTP; **(A)** Overall; **(B)** Treatment Line 1; **(C)** Treatment Line 2; and **(D)** Treatment Line 3.

Based on Kaplan–Meier estimates, the median overall TTP was 31.3 months (95% CI: 10, 52.6). Whereas the median TTP was 23.9 (95% CI: 19.1, 28.8) months following the 1st line of treatment, 10.1 (95% CI: 4.8, 15.5) months following the 2nd line of treatment, and 5.9 (95% CI: 1.2, 10.6) months following the 3rd line of treatment (**Figure 1**).

**Figure 2:**
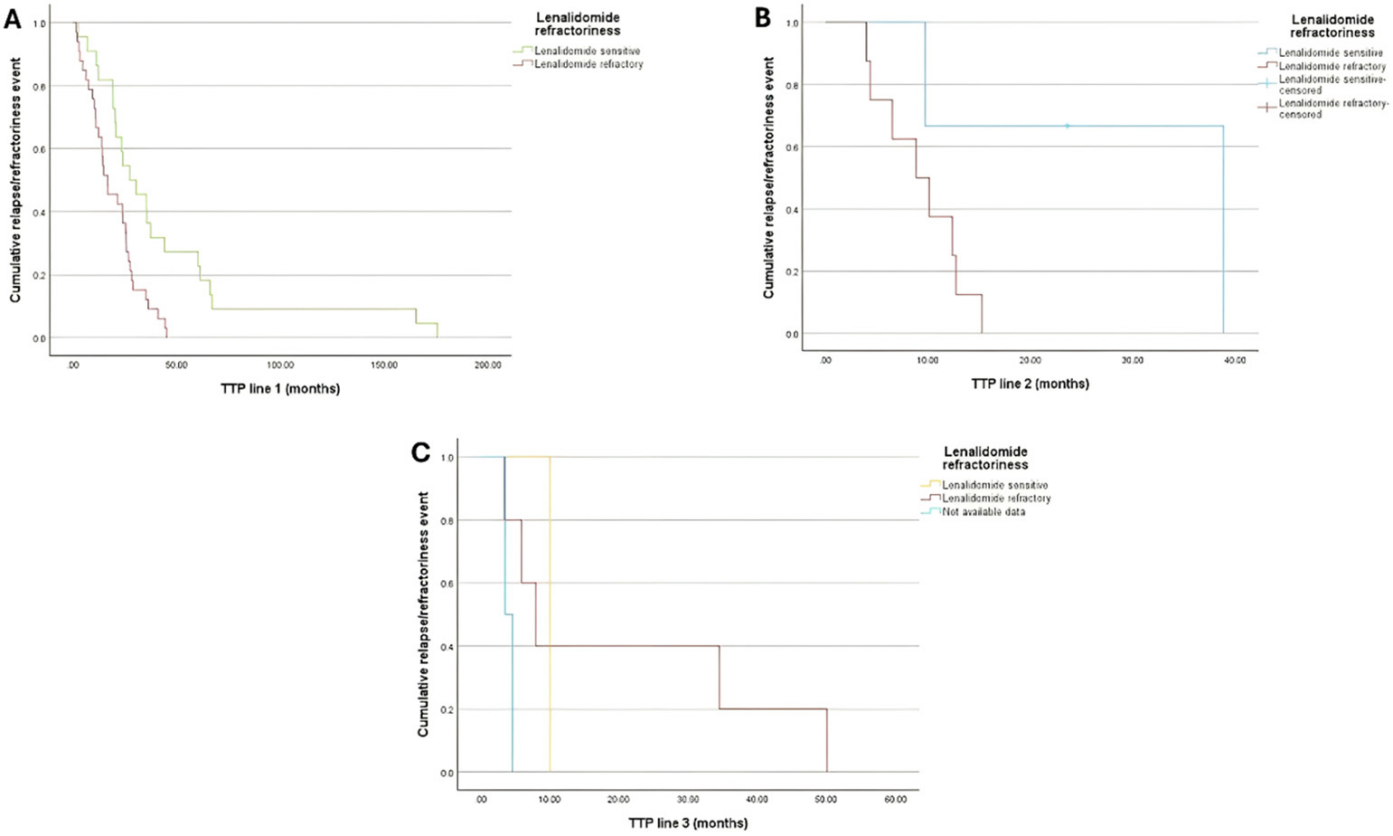
Kaplan-Meir curve for TTP of patients with lenalidomide refractoriness (Either being lenalidomide-refractory or lenalidomide-sensitive) by the end of **(A)** Treatment Line 1; **(B)** Treatment Line 2; and **(C)** Treatment Line 3.

The median TTP was 16.7 (95%CI:8.6, 24.8), 8.9 (95%CI: 3.9, 13.9), and 8 (95%CI: 3.5, 12.4) months among lenalidomide refractory patients compared to 27.3 (95%CI:13.5, 41.2), 38.8, and 10 months lenalidomide-sensitive patients following the 1^st^, 2^nd^, and 3^rd^ lines of treatment, respectively (**Figure 2**).

### Renal impairment and respiratory complications among participants

Among patients receiving the first, second, third, and fourth lines of therapy, the proportions of patients with renal impairment were 23.6%, 17.9%, 20.3%, and 6.3%, respectively. Additionally, respiratory complications, including asthma and/or COPD, were detected in 10.1%, 10.1%, 18.2%, and 18.8% of patients receiving the 1^st^, 2^nd^, 3^rd^, and 4^th^ lines of treatment, respectively (**Table 4**).

**Table 4:** Proportion of patients with renal impairment and asthma and/or COPD.

Type of impairment	N (%)
Renal impairment	
At 1st line (N=148)	35 (23.6%)
At 2nd line (N=139)	25 (17.9%)
At 3rd line (N=54)	11 (20.3%)
At 4th line (N=16)	1 (6.3%)
Asthma and/or Chronic obstructive pulmonary disease (COPD)	
At 1st line (N=148)	15 (10.1%)
At 2nd line (N=139)	14 (10.1%)
At 3rd line (N=54)	10 (18.2%)
At 4th line (N=16)	3 (18.8%)

*1st line Vs. 2nd line: P. Value>0.05, 2nd line Vs. 3rd line: P. Value<0.05, 3rd line Vs. 4th line: P. Value<0.05.*

Renal improvements were observed among 51.4%, 52%, 54.5%, and 100% of patients receiving the 1^st^, 2^nd^, 3^rd^, and 4^th^ lines of therapy, respectively. The proportion of patients who showed improvement in renal impairment after the 1st, 2nd, 3rd, and 4th lines of therapy was 51.4%, 52%, 54.5%, and 100%, respectively, out of the number of patients with renal impairment at each line. The mean eGFR was 40.5 (48.8) at baseline, 47 (38.2) after the 1st line, 63.5 (48.8) after the 2nd line, and 73 (15.5) after the 3rd line of therapy. However, there was no statistically significant difference in eGFR compared to baseline.

Regarding respiratory complications, including asthma and/or COPD, improvements were only observed in 20%, 7.1%, and 40% of patients receiving the 1^st^, 2^nd^, and 3^rd^ lines of therapy, respectively. The rate of improvement of respiratory complications was not statistically significant across all the treatment lines, except between the 2^nd^ and 3^rd^ lines of therapy (p<0.05) (**Supplementary Table S5**).

### Rates of Response to Various Treatment Lines

About 43.2%, 33.1%, 16.6%, and 6.2% achieved CR following the 1^st^, 2^nd^, 3^rd^, and 4^th^ lines of treatments, respectively. PR was achieved in 13.5%, 10.1%, and 14.8% after receiving the 1^st^, 2^nd^, and 3^rd^ lines of treatments, respectively. Meanwhile, VGPR was attained at 17.6%, 13%, 12.9%, and 6.2%, respectively. However, disease progression occurred in 12.2%, 13%, 20.3%, and 12.5% among those treated with the 1^st^, 2^nd^, 3^rd^, and 4^th^ lines of treatments, respectively (**Supplementary Table S6**).

### The minimal residual disease status across different lines of therapy

MRD negativity was detected in 11.5%, 7.2%, and 1.8% following the 1st, 2nd, and 3rd lines of treatment, respectively (**Supplementary Table S7**).

### The frequency of isatuximab-based regimens

Here, isatuximab was used in 20 patients. Among them, 80% were used in the 2nd line, 15% in the 3rd line, and 5% were administered in the 4th line of treatment (**Table 5**).

**Table 5:** Isatuximab use among participants.

Isatuximab	N (%)
**1st Line**	0 (0%)
**2nd Line**	16 (80%)
**3rd Line**	3 (15%)
**4th Line**	1 (5%)

## Discussion

We retrospectively reviewed patient demographic and clinical characteristics, treatment lines, and outcomes in RRMM patients in the Greater Gulf region. Our findings highlight the aggressive nature of MM, with high relapse rates and increased refractoriness to treatment over successive lines of therapy. As disease control becomes challenging over time, there is a critical need for effective therapeutic strategies to improve long-term outcomes.

Among 148 patients eligible for the study, the mean age was 59.4 years old. This is comparable to the median age reported in several studies in the Middle East and North Africa (MENA) region, which reported a median age of 61.98 (24) in Iran, 61.91 in Lebanon (25), and 58.74 years in Saudi Arabia (26). In newly diagnosed MM patients in Saudi Arabia, the reported age was 56 (27), and 51 (28) Years old in another two studies. However, this is younger than that reported in international data (29–32). This difference may be attributed to the early detection and demographic differences. Additionally, males were more likely to be diagnosed with RRMM, which is consistent with the literature (28, 33–36), though females had a higher risk of adverse molecular risk lesions (37), and worse PFS if diagnosed under 50 (38).

SCT is considered a preferred treatment option for MM patients who have received initial treatment and are eligible for transplant. Moreover, incorporating pre- and post-treatments, especially novel therapies, has led to deeper responses and enhanced PFS (39). According to recent National Comprehensive Cancer Network (NCCN) guidelines, VRd or KRd (Carfilzomib, lenalidomide, and dexamethasone) are the preferred regimens as induction therapy among transplant-eligible patients (40). Other recommended regimens include DVRd (40).

Here, SCT was performed for 85.9% of the patients. This high rate is explained by the high proportion of patients younger than 65 years old in our cohort. Moreover, CR was achieved in 47.76% of patients, and VGPR and PR were achieved in 14.93% and 11.94% of participants, respectively. The VRd and VCd combinations were mostly used as induction and consolidation therapy. While a combination of Rd was the most common maintenance regimen. MRD negativity was detected in 70.59% of the patients who underwent MRD testing post-SCT.

Paquin et al. reported that the 4-year OS among transplant-eligible patients aged <65 years was 82%, with no significant difference in OS by the timing of the transplant or the initial regimen administered (41). However, a meta-analysis by Jain et al. revealed that early SCT and the use of novel agents as induction therapy were associated with improved PFS, increased response rate, and CR rate but with no OS benefits, suggesting that further evaluation of the clinical utility and beneficial combination induction regimens is still needed (42).

Comparing VRd and VCd, VCd was associated with a lower CR rate, but there was no significant difference between either regimen regarding VGPR, OS, and PFS. Moreover, patients receiving VRd regimens showed a higher rate of renal recovery (43, 44). A meta-analysis by Yang et al. reported that VRd induction therapy resulted in 91%, 23%, and 56% overall response, VGPR, and CR rates, respectively. VRd led to a better CR rate compared to VCd and prolonged 1- and 3-year OS compared with VTd (45).

In a real-world experience in Lebanon, VCd was the most commonly used induction protocol, followed by VTd and VRd, and there was no significant difference between the three regimens regarding the OS and PFS (25). Still, the PFS was significantly higher among patients who underwent SCT than those who did not (25). In a retrospective study in Saudi Arabia, VAd (Vincristine, Doxorubicin, and Dexamethasone) and VCd were the most frequently used induction therapy, and the post-induction CR rate was 50% and increased to 78.1% following the transplantation (28). Additionally, a clinical trial by Sonneveld et al. addressed the role of VRd consolidation therapy followed by continuous lenalidomide maintenance therapy and found that the use of the VRd consolidation regimen was associated with improved CR rate and PFS with acceptable toxicity compared to maintenance alone, suggesting that VRd regimen was a feasible consolidation regimen (46). On the contrary, Stadtmauer et al. reported no significant improvement in PFS or OS following VRd consolidation therapy or second autologous SCT (47).

Bortezomib- and lenalidomide-based maintenance regimens have been shown to prolong PFS, OS, TTP, and time to next treatment (TNT). Bortezomib-based regimens would be more feasible compared to lenalidomide-based regimens in resource-limited settings, high-risk patients, those with renal insufficiency, those with a lack of tolerance to lenalidomide, or those with a previous history of cancer. Therefore, the choice of maintenance regimen should be personalized (48–59).

The lack of significant OS benefits of these drug combinations in different trials might be due to several factors, including the availability of multiple effective treatment regimens over the past years, differences in patients’ characteristics, variable SCT strategies, diverse treatment modalities, and duration of therapy, the use of maintenance therapy, and short follow-up periods.

Among ineligible patients for SCT, VRd was the most frequently administered first-line treatment (40.2%), followed by the VCd regimen (20.7%). Also, CR was observed in 43.2% of the participants, while PR and VGPR were detected in 13.5% and 17.6% of the patients, respectively. MRD negativity was detected among 11.5% of the patients.

The efficacy and tolerability of VRd as first-line therapy among newly diagnosed MM patients have been well established in an open-label phase 1/2 trial, marking it as the first regimen to result in a 100% response rate (60). The SWOG S0777 trial confirmed that VRd resulted in an improvement in the rate of response, depth of response, PFS, and OS among transplant-ineligible newly diagnosed MM patients (61). Nevertheless, in real-world experience studies including patients treated with first-line VRd, older age, having high-risk cytogenetics, advanced tumor stage, and worse ECOG performance status score were associated with increased risk of disease progression/death (62). When comparing VRd with VCd, VRd was associated with a higher response rate, longer PFS, and improved OS than VCd (63).

Here, the DKd combination regimen was the most commonly used second-line treatment, followed by the DVd, DRd, and Isa-Pd regimens. Additionally, after the 2^nd^ line treatment, we observed that the CR, PR, and VGPR rates were 33%, 10%, and 13%, respectively. MRD negativity was detected among 7.2% of the participants. Furthermore, 54 patients received third-line treatment regimens. Among them, KPd was the most widely prescribed, followed by DKd, DPd, and DRd. Furthermore, the rate of CR, PR, VGPR, disease progression, and MRD negativity was 16.6%, 14.8%, 12.9%, 20.3%, and 1.8%, respectively.

A clinical trial indicated that Kd (Carfilzomib and Dexamethasone) resulted in significant improvements in survival outcomes along with the reduction of death compared to Vd (Bortezomib and Dexamethasone) among RRMM patients (64). Furthermore, DKd was associated with a reduced risk of disease progression/death, deeper response, increased MRD negativity, and maintained survival benefits compared to KD alone in RRMM patients, including lenalidomide-refractory MM patients (65–67). These findings were further supported by the IKEMA trial, which demonstrated that isatuximab-based regimens significantly improved PFS and OS in patients with RRMM regardless of prior lenalidomide exposure (68, 69).

Lenalidomide is the most prescribed drug among MM patients, which is widely incorporated in many therapeutic regimens. However, despite its significant efficacy, there is a growing resistance to lenalidomide (70–72). Our findings on lenalidomide refractoriness align with existing literature, which reports significant resistance rates and advocates for lenalidomide-sparing regimens to address this challenge in MM treatment (69).

In our study, renal impairment was detected more frequently in MM patients treated in their NDMM stage. Chen et al. (73), and Courant et al. (74), have reported that severe renal impairment was a prognostic factor for poor survival outcomes among newly diagnosed MM patients after 6 months of diagnosis. Moreover, bortezomib and novel agents-based regimens were safer and more effective options for patients with MM and renal impairment without the need for dose modifications (75). Additionally, daratumumab plus dexamethasone and Isa-Pd regimens were shown recently to have a deeper response and improved survival with favorable safety profiles among RRMM patients with renal impairment or on dialysis (76–78). Multiple studies have demonstrated that Isa-Pd and Isa-Kd significantly improved PFS in patients with RRMM, including those with moderate to severe renal impairment. For instance, Capra et al. reported a median PFS of 13.4 months for patients with RI treated with Kd, compared to a median PFS that was not reached for patients treated with Isa-Kd (hazard ratio [HR]: 0.27; 95% confidence interval [CI]: 0.11-0.66) (79). Additionally, Dimopoulos et al. found a median PFS of 9.5 months for patients with RI treated with Isa-Pd (n=55), compared to 3.7 months for those treated with Pd (n=49; HR: 0.50; 95% CI: 0.30-0.85) (78). As this is a retrospective study, we do not have specific data on the reasons for the observed trend in renal impairment. However, one possible explanation is that patients with significant renal dysfunction may have been unable to tolerate further lines of therapy and were therefore underrepresented in the later treatment groups. Additionally, the smaller sample sizes in the third and especially the fourth-line groups may also contribute to variability in these percentages.

Notably, we observed asthma and/or COPD among 10.3%, 10.07%, 18.15%, and 18.75% of patients receiving the 1^st^, 2^nd^, 3^rd^, and 4^th^ lines of treatment, respectively, which significantly improved with shifting to new lines of treatment. Rice et al. observed about 15% of MM patients had asthma or COPD at diagnosis, and the most used agents were lenalidomide and bortezomib (4). These patients had a significantly prolonged time from first-to-second-line treatment but worse survival outcomes compared to those without asthma or COPD with a high probability of treatment discontinuation (4). There are limited studies, including patients with COPD/asthma receiving biologic treatment due to concerns regarding the possible adverse effects of monoclonal antibodies.

Here, out of the 20 patients who used isatuximab, 80% of cases were used in the 2^nd^ line, 15% in the 3^rd^ line, and 5% in the 4^th^ line of treatment. Early studies of Isa-Pd in RRMM patients found that the 10 mg/kg isatuximab resulted in a clinically meaningful response and accepted safety profile (80). Furthermore, in phase 3 trials, Isa-Pd achieved an improved overall response rate, PFS, and MRD negativity rate (11, 81). Recent real-world data further support isatuximab-based regimens efficacy, with Isa-Kd achieving an 85% overall response rate and a short time to best response (82), while Isa-Pd has shown promise as a valuable option for patients refractory to daratumumab (83).

The efficacy and safety of isatuximab 10 mg/kg combined with pomalidomide and dexamethasone or with carfilzomib-dexamethasone among RRMM patients were proven in the ICARIA-MM (11) and IKEMA (69) trials, respectively. Promising findings of incorporating isatuximab in different 1^st^ line treatment regimens for newly diagnosed MM were also confirmed (84–86).

A recent meta-analysis concluded that combining anti-CD38 monoclonal antibodies with PIs (or IMiDs) and dexamethasone significantly improved OS and PFS in RRMM patients. This combination also achieved higher rates of overall response, complete response or better, VGPR or better, and MRD-negative status compared to using PIs (or IMiDs) and dexamethasone alone. These results highlight the impact of incorporating anti-CD38 monoclonal antibodies into treatment regimens for enhancing patient outcomes in RRMM (15).

It is important to note that different factors, including the number of previous treatment lines, the type and length of the administered treatment regimens, and baseline high-risk cytogenetic profile would influence the efficacy of different regimens among RRMM patients (87).

## Conclusion

This retrospective real-world study highlighted the large diversity of treatment regimens across MM patients at different relapsing/refractoriness stages in the Gulf Region. The study outcomes offer valuable insights into the practical clinical advantages of using SCT and different regimens of various lines of treatment in RRMM. These benefits extend to lenalidomide-refractory patients, with efficacy rates similar to those observed in controlled clinical trials. These findings can help guide the development of future treatment protocols to enhance outcomes for RRMM patients.

## Data Availability

The original contributions presented in the study are included in the article/**Supplementary Material**. Further inquiries can be directed to the corresponding author.
