# Supplementary material for: Analysis of pharmacotherapeutic approaches for multiple myeloma and correlated renal and pulmonary impairments: a retrospective real-world registry study in the Greater Gulf Region (REPAIR Study)
Source: Front Oncol. 2025 May 9;15:1547138. doi: 10.3389/fonc.2025.1547138 (PMC12098271; doi:10.3389/fonc.2025.1547138)
Supplement: Supplementary file 1 [file DataSheet1.docx]

**Supplementary material**


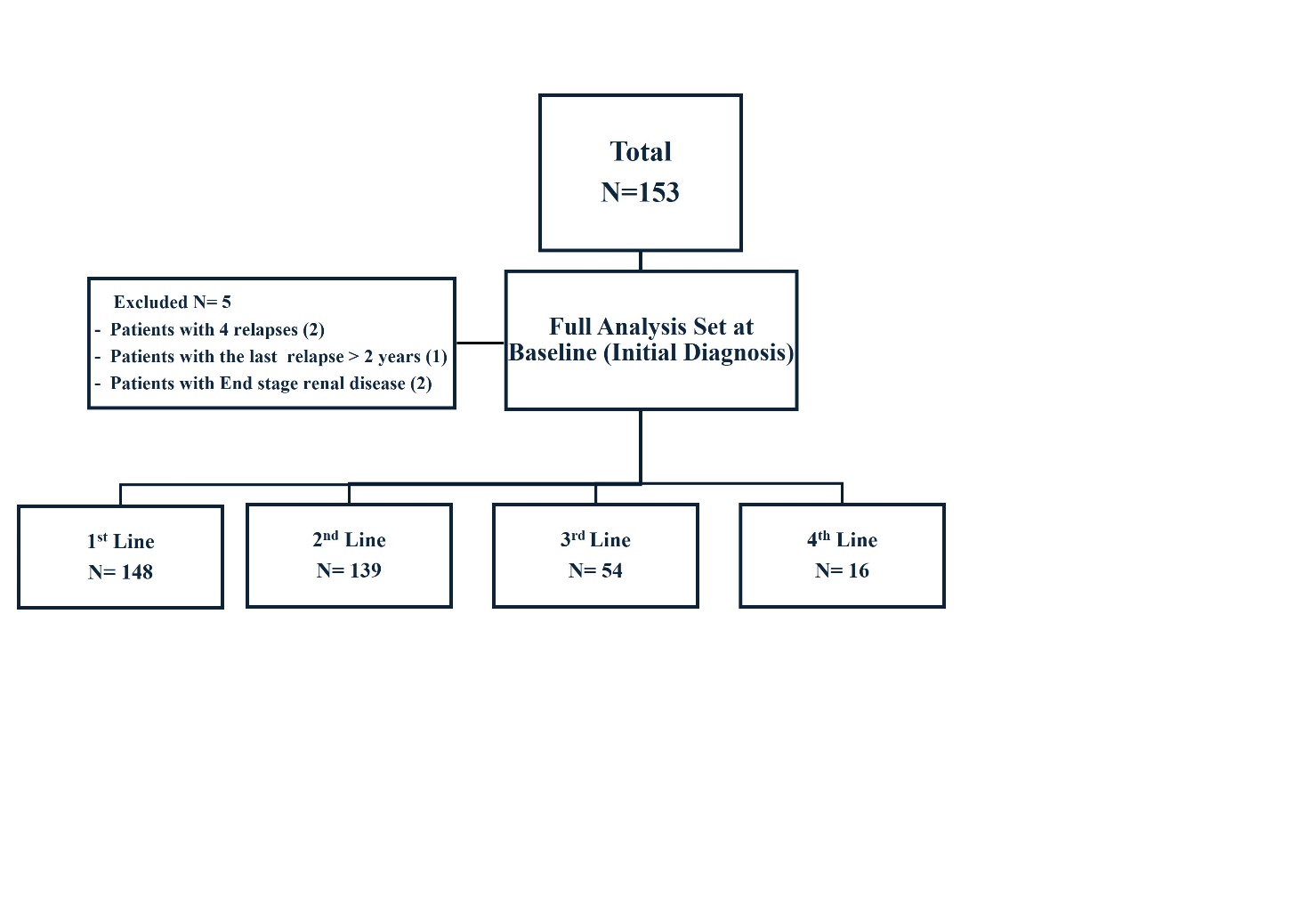


**Supplementary Figure 1:** Patient Disposition in the Study

**Supplementary Table 1.** Multiple Myeloma Disease History at Baseline

| **MM Disease History** | **Values** |
| --- | --- |
| **Disease duration in months, Median (Range)** | 35 (2-246) |
| **Baseline diagnosis, N (%)**  MM  MGUS  MM, Solitary plasmacytoma  Solitary plasmacytoma  Systemic AL amyloidosis | 142 (95.9%)  1 (0.7%)  2 (1.4%)  2 (1.4%)  1 (0.7%) |
| **Clinical stage at diagnosis – as per ISS, N (%)**  Stage I  Stage II  Stage III  Pre-stage MM  NA | 10 (6.8%)  39 (26.4%)  59 (39.9%)  1 (0.7%)  39 (26.3%) |
| **Age at time of diagnosis in years, Median (Range)** | 56 (18-84) |

***Abbreviations****: MM: multiple myeloma, MGUS: monoclonal gammopathy of unknown significance, ISS: International staging system, NA: Not Available.*

**Supplementary Table 2**: Status of Stem Cell Transplantation Among Participants

|  | **N (%)** |
| --- | --- |
| **Was the subject eligible for SCT?**  Yes  No  Not available | 78 (52.7%)  51 (34.5%)  19 (12.8%) |
| **Reasons for ineligibility of SCT^*^**  Advanced age  Comorbid conditions  Patient refusal  Other causes (e.g., suboptimal response, age, or progressive disease) | 22 (43.3%)  11 (21.6%)  14 (27.5%)  10 (19.6%) |
| **Number of SCTs performed** | 67 (85.9%) |
| **Type of SCT performed**  Allogeneic  Autologous  Not available | 1 (1.3%)  66 (84.6%)  11 (14.1%) |
| **Sources of SCT**  Bone marrow  Peripheral  Cord stem  Not available | 15 (19.2%)  48 (61.5%)  1 (1.3%)  14 (17.9%) |
| **Response to SCT**  Stringent Complete response  Complete Response  Very Good Partial Response  Partial Response  Minimal Response  Disease Progression  Not Evaluable  Not Available | 5 (7.5%)  32 (47.8%)  10 (14.9%)  8 (11.9%)  2 (3%)  6 (9%)  2 (3%)  2 (3%) |
| **MRD Status post SCT**  Negative  Positive  Total MRD-tested patients  Not Performed  Not Available | 12 (70.6%)  5 (29.4%)  17 (100%)  48  13 |

**Patient may have more than one reason for ineligibility*

***Abbreviations****: SCT, Stem cell transplant; CYC, Cyclophosphamide; MRD, Minimal residual disease.*

**Supplementary Table 3**: Relapse/Refractoriness Following Different Treatment Lines.

| **Line of treatment** | **Refractoriness, N (%)** | **Relapse, N (%)** |
| --- | --- | --- |
| **1st line** | 2 (1.3%) | 144 (98.6%) |
| **2nd line** | 10 (16.1%) | 52 (83.8%) |
| **3rd line** | 9 (34.6%) | 17 (65.3%) |

**Supplementary Table 4.** Time to Disease Progression and Duration of Response Among the Participants.

|  | **Median (Range)** |
| --- | --- |
| **TTP in months from the beginning of the line till DOP**  1st line  2nd line  3rd line  4th line  Overall | 23.6 (1.5-175.6)  11.3 (4-38.8)  6.9 (3.4-50)  2.5 (0.2-12.8)  36.9 (20.4-244.8) |
| **DoR in months^*^**  1st line  2nd line  3rd line  Overall | 17.2 (0.03-171.8)  7.5 (0.1-65.1)  4.4 (0.5-44.7)  23.8 (20.3-238.7) |

**DoR: The time of first recorded achievement of a particular response level [Date of relapse – Date of response]*

***Abbreviations****: TTP, Time to Progression; DOP, Date of Progression; DoR, Duration of Response.*

**Supplementary Table 5:** Improvement of Renal and Pulmonary Impairment among Patients Following Different Lines of Treatment

|  | **Values** | **P value** |
| --- | --- | --- |
| **Improved renal impairments, N (%)**  1st line  2nd line  3rd line  4th line | 18 (51.4%)  13 (52%)  6 (54.5%)  1 (100%) | **<0.05** |
| **Improved pulmonary impairments, N (%)**  1st line  2nd line  3rd line | 3 (20%)  1 (7.1%)  4 (40%) | **<0.05 only in 2nd line Vs. 3rd line** |
| **Change of eGFR from the baseline, Mean (SD)**  Baseline (Initial diagnosis)  After 1st line  After 2nd line  After 3rd line | 40.5 (48.8)  47 (38.2)  63.5 (48.8)  73 (15.5) | **0.407** |

**Supplementary Table 6**: Treatment Outcomes for Each Lines of Treatment

| **Treatment outcomes** | **N (%)** |
| --- | --- |
| **1st line treatment outcomes**  Complete Response  Disease Progression  Minimal Response  Not Evaluable  Partial Response  Stable Disease  Stringent Complete response  Very Good Partial Response  Not Available | 64 (43.2%)  18 (12.2%)  4 (2.7%)  4 (2.7%)  20 (13.5%)  4 (2.7%)  7 (4.7%)  26 (17.6%)  1 (0.7%) |
| **2nd line treatment outcomes**  Complete Response  Disease Progression  Minimal Response  Not Evaluable  Partial Response  Stable Disease  Stringent Complete response  Very Good Partial Response  Not Available | 46 (33.1%)  18 (13%)  5 (3.6%)  23 (16.6%)  14 (10.1%)  4 (2.9%)  4 (2.9%)  18 (13%)  7 (5.0%) |
| **3rd line treatment outcomes**  Complete Response  Disease Progression  Minimal Response  Not Evaluable  Partial Response  Stable Disease  Stringent Complete response  Very Good Partial Response  Not Available | 9 (16.6%)  11 (20.3%)  1 (1.8%)  11 (20.3%)  8 (14.8%)  2 (3.7%)  1 (1.8%)  7 (12.9%)  5 (9.2%) |
| **4th line treatment outcomes**  Complete Response  Disease Progression  Minimal Response  Not Evaluable  Stable Disease  Very Good Partial Response | 1 (6.2%)  2 (12.5%)  1 (6.2%)  9 (56.2%)  2 (12.5%)  1 (6.2%) |

**Supplementary Table 7**: Minimal Residual Disease (MRD) Status

| **MRD Status at different lines of treatment** | **N (%)** |
| --- | --- |
| **1st line**  Positive  Negative  Not Performed  Not Available | 19 (12.8%)  17 (11.5%)  110 (74.3%)  2 (1.4%) |
| **2nd line**  Positive  Negative  Not Performed  Not Available | 10 (7.2%)  10 (7.2%)  117 (84.2%)  2 (1.4%) |
| **3rd line**  Positive  Negative  Not Performed | 4 (7.4%)  1 (1.8%)  49 (90.7%) |
| **4th line**  Positive  Not Performed | 1 (6.3%)  15 (93.7%) |
